# Progress in Medicine

**Published:** 1897-04-10

**Authors:** 


### Progress in Medicine.

### RHEUMATISM AND GOUT.

(*Continued from page 13.*)

Rheumatoid arthritis is another of the diseases often classed with true rheumatism. G. A. Bannatyne, who has done so much work on its ætiology,[1] remarks that anæmia is seldom very marked except in the severest cases, though a slight amount is a very common characteristic. The number of the red corpuscles is decreased much less than their hæmoglobin; there are many of altered shape and size, but no nucleated ones, while the white corpuscles are somewhat increased in number. Similarly, in malarial diseases there is a special loss of the hæmoglobin, while in true rheumatism the hæmoglobin value of each corpuscle is not diminished, and in pernicious anæmia it is even increased. Whether these differences depend on the toxins in each case or on other factors is not clear. Potani,[2] in considering the production of the deformities, considers that they are due not to alterations in the bones, but rather to weakening of the muscles through a sort of reflex degeneration, so that their balance is disturbed. Osseous changes are of less importance, and often are rather the results of the change of direction rather than causes.

**Treatment.**—Baker[3] discusses the methods now employed in rheumatism. In the acute form he prefers salicylate of soda till the pain vanishes or tinnitus sets in, when reduced doses of salicin may be used. Iodide of potash may be added in chronic cases, and where the pain is worse at night quinine and iodide of potash yields good results. Cappelari[4] considers the effect of salophen in sciatica as marvellous, removing the pain in cases where other remedies had been tried in vain. He employs doses equal to one drachm daily with no ill effects. Norström[5] distinguishes in sciatica not only the forms due to neuralgia and neuritis, but also an accompanying myositis which may affect the upper fibres of the glutens medius and the peroneal muscles, especially their lower part. This myositis of rheumatic origin may at first occur without the nerve being affected. It is important to recognise this inflamed muscle, since treatment by massage will often bring about a cure, while remedies directed to the neuralgia alone fail. The massage must be applied in the exact direction of the fibres of the diseased muscle. C. Heldenburgh[6] holds that typical lumbago, arising suddenly during an effort, and showing severe pain, increased by the slightest movement, is really a sprain of the sacro-vertebral articulation. The pain can be readily produced by pressure of the thumb on the joint, and relief is best obtained by placing the patient on his back with a hard pad under the joint, and instructing him to go through a series of exercises of flexion and extension of the lower limbs on the trunk. Malakine is recommended as a substitute for salicylates by Merkel,[7] who finds that it acts well without causing tinnitus. Serum from convalescents was injected into ten patients by Jules Weiss,[8] with the idea that if rheumatism is a microbic infection antitoxins would be present. In several cases salicylates had to he finally used; but in some definite relief followed the employment of the serum. E. H. King[9] aims at increasing the elimination of uric acid and other poisons, and therefore gives a preliminary dose of calomel and salines before the salicylates. The value of the latter and of iodide of potash depends, he thinks, largely on the increased excretion of urea caused by them. The hot-air local bath treatment for rheumatic and other stiff and painful joints has been employed by V. P. Gilney,[10] who reports considerable success. The joints were exposed to a heat of about 250 deg., and much improvement followed, both as to pain and movement. Another case is described by Knowsley Sibley,[11] who brought forward so many brilliant results last summer. The patient had not seen the palms of her hands for two years, but she was able to do this after three applications. The right knee was quite immovable, but this and other serious lesions were quickly relieved.

Professor Neusser,[12] in experimenting with Ehrlich's stain for the leucocytes of the blood, to which some methyl green had been added, found that spots of pigment developed close to the nuclei in patients sufferin from lithæmia and uric acid diseases such as gout. This sign, called Neusser's Basophily, is a fairly constant indication of these affections, and especially important in the diagnosis of cases where gout is suspected, for where gout exists this indication seems to be universally present. Neusser remarks that where these granules are found in phthisical patients we can predict that the disease will take the chronic fibroid form due to the modifying influence of gout. It is said, however, by some that these granules can be found in most leucocytes,[13] while Kolisch thinks that the uric acid excreted in the urine varies directly with the number of these granules. Futcher, however, disproves this relationship if his experiments are correct.

A useful review of the work done upon uric acid of late years is given by A. Mathews,[14] showing that the greater part of the uric acid in birds and the formation of urea in mammals is brought about by the synthesis of ammonia with unknown bodies through the agency of the liver; but the uric acid found in mammals, and possibly a small part of that in birds, is derived from the decomposition of cell nuclei, and chiefly from those of leucocytes. The latter proposition is illustrated by the increase of uric acid when leucocytosis occurs, as after flesh meals, in leucocythæmia, in phosphorus poisoning acute fevers, and burns; so, too, after the administration of pilocarpine, thymus gland, nuclein, or aseptic pus, more uric acid is excreted. Haig,[15] of course, insists that much of the uric acid is derived simply from that taken in as food. Thus by eliminating from the diet all animal foods except milk and cheese, as well as tea and coffee, a decided reduction of the uric acid can be effected. Yet, as stores of this substance may remain in the body for a year or more, a vegetarian diet may be necessary for this period before the relation of uric acid to urea can be brought down permanently to less than one in thirty. Under this treatment his charts show uric acid falling from about four grains to less than one daily. To find out whether any stored-up acid still exists, he gives three grammes of salicylate of soda. A great increase of uric at once follows if there is any stored up. If there is no rise he concludes that the tissues have been swept clean. In this way he would guard against high tension pulse, headache, gout, rheumatism, albuminuria, and epilepsy. Uric acid disorders depend not on any excessive production, but simply on the amount introduced in food.[16] Vaughan Harley and others reply that in children there is much more uric acid excreted per unit of body weight than in adults on the same diet, and that a leucocythæmic patient will excrete several times the amount given off by a healthy man under similar conditions.

J. Hutchinson[17] has views of his own on gout. He believes that there is always rheumatism present in gout, and nearly always an hereditary taint. Patients must beware of fruit, especially gooseberries, and of alcohol, especially that to which grape sugar has been added. Th. Schott[18] considers that gout directly affects the heart, and that urates can be demonstrated in sclerosed aortic valves by the murexide reaction. It also assists other factors in the production of valvular disease. Still more frequently it produces motor and sensory cardiac neuroses, palpitation, tachycardia, dilatation, and pains of various kinds. There has been much discussion on the value of piperazin and lysidin, and some light has been thrown on the question by the researches of F. W. Goodbody.[19] He chose as his method the examination of the power these drugs have of keeping urates in solution in the urine, since uric acid always exists in the blood, and is excreted in some form of urate. The urine of a patient suffering from uric acid gravel was the subject of the tests, and great care was taken to avoid all sources of error. The drugs were given internally and added to the urine in separate experiments. The results show that either drug, when added to the urine, is capable of hindering deposit, lysidin being the more powerful solvent. Both drugs when given internally increase the elimination without increasing its formation in the body, so that after a time there is a decreased amount excreted. Both are diuretics, and increase the elimination of nitrogen. As to general treatment of gout, C. C. Ransom of New York,[20] advises the administration of large quantities of pure water in preference to alkaline draughts, together with hepatic stimulants and mild purgatives, hot baths from sulphur and other springs, modified to prevent depressing effects, to the number at least of 20. He allows meat once a day (except veal and pork), and even twice if desired; sweets are restricted, and potatos and other roots forbidden; but the wants of each patient must be studied. In chronic gout he gives colchicum and iodide of potash for weeks together after an initial calomel purge. Careful massage of the joints, an ointment containing iodide of potash and iodine, and where there is much pain icthyol ointment of 30 to 50 per cent. are recommended. In acute attacks an initial dose of calomel is also desirable, to be followed by 15 minim doses of colchicum. A liquid diet of milk, broth, and gruel is maintained until the patient is able to get about. In giving the course of baths it is important to reduce the temperature and duration of the bath even to five minutes or less if the patient is unfavourably affected, but they should be employed daily. Most patients, however, suffer during the first week of bathing from a "bath fever," headache, coated tongue and digestive disturbance. The bath may be then omitted for a day or two, and a dose of calomel given, which always removes the symptoms.

[1] Lancet, Nov. 28. [2] Med. Week, Dec. 18. [3] Therap. Gazette, Oct. 15. [4] Clin. Jour., Feb. 10. [5] New York Med. Jour., Dec. 17. [6] Med. Week, Feb. 5. [7] Therap. Gazette, Oct. 15. [8] Amer. Jour. Med. Sci., Oct. [9] Jour. Practic. Med., No. 11. [10] Med. Record, Jan. 23. [11] B.M.J., Nov. 21. [12] Med. Surg. Bulletin, Nov. 14. [13] Edin. Med. Jour., Nov. [14] New York Med. Jour., Oct. 31. [15] B.M.J., Oct. 3. [16] Lancet, Nov. 28. [17] Clin. Jour., Sept. 30. [18] Berlin Klinic Woch., Nos. 21, 23. [19] B.M.J., Oct. 3. [20] Med. Rec., Feb. 6.

